# There Is Always Something New Out of Africa

**DOI:** 10.3201/eid1607.AC1607

**Published:** 2010-07

**Authors:** Polyxeni Potter

**Affiliations:** Author affiliation: Centers for Disease Control and Prevention, Atlanta, Georgia, USA

**Keywords:** Art-science connection, emerging infectious diseases, art and medicine, Prince Twins Seven-Seven, *The Lazy Hunters*, *and the Poisonous Wrestlers*, *Lizard Ghost and the Cobra*, Nigerian art, African art, about the cover

**Figure:**
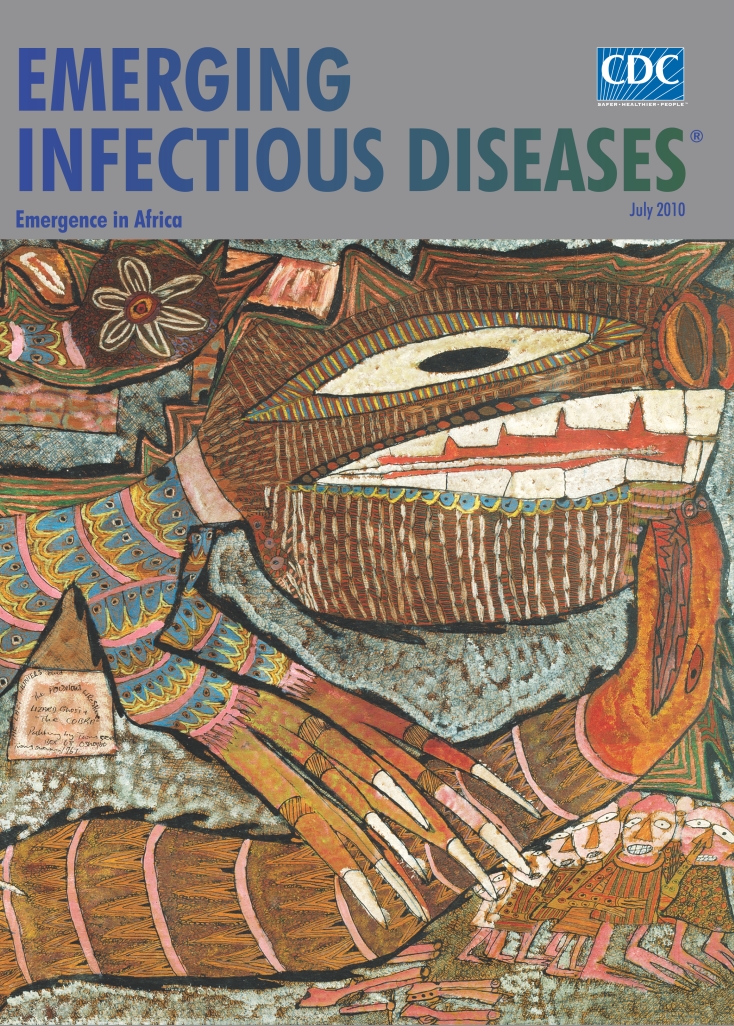
**Prince Twins Seven-Seven (b. 1944). *The Lazy Hunters, and the Poisonous Wrestlers, Lizard Ghost and the Cobra* (1967)**. Ink, paint, and chalk on plywood (124.5 cm × 78.1 cm) , National Museum of African Art, Washington, DC, USA. Gift of Mr. and Mrs. Sean Kelly, 75–28–3

—Pliny the Elder, Natural History (c. 2,000 years ago)

Lone survivor of seven sets of twins born to his mother, he started life as a legend among the Yoruba in Nigeria, who have one of the highest rates of twin births and infant deaths in the world. According to family lore, he was the spirit that kept trying to be born and kept being turned back, the child “born to die.” Bamidele to his father, Olaniyi to his grandmother, Prince Twins Seven-Seven renamed himself in adulthood to commemorate newly found royal lineage and the circumstances of his birth, which colored his life and shaped his art. Deeply conscious of these aspects of his nature, he has conveyed in art his personal understanding of a world teeming with spirits and populated with myths.

A young man in his early 20s, he went on the road as a dancer and musician. “I was born with music.” He found himself in the town of Oshogbo, in the heart of southwestern Nigeria’s Yoruba-speaking people. “I was…doing entertainment for traders to boost their sales. They would hire me and I would dance to attract people.” Oshogbo was home to a namesake artists group, progenitor of modernism. German scholars Ulli and Georgina Beier promoted literature and the arts there and conducted workshops for young talent outside the academic art scene. Twins was invited to stay and soon showed artistic promise with imaginative works free of form, perspective, proportion, or convention. “I don’t want to do what everybody does.” From the start, his works were instead vibrant and rich in linear and color motifs and were informed by the surrounding culture, one of the oldest, largest, and most influential in Africa, with a long tradition of sculpture, wood and metal carving, textiles, and beadwork.

The Yoruba acknowledge a supreme deity, Olorun, and view the universe as two halves of a closed calabash. One filled with living beings―animals, people, and plants; the other with spirits, deities, and ancestors connected with natural forces―thunder, rain, disease. When the sky god sent his son to earth, the son became the first ruler of the Yoruba kingdom. As his direct descendants, the first kings and their offspring became divine kings. In this world of complicated and constant interaction, living beings draw on surrounding forces to realize their personal destiny. Their descendent artist does no less. Steeped in these traditions, he brings through his art their authenticity and purity to the world.

“In order to make people look, you have to make it extraordinary.” Twin’s interpretation of this tightly woven cultural fabric had immediate and wide popular appeal. His works, usually intense personal variations of Yoruba myth often interspersed with his own life story, were well received at home and abroad and inspired many followers and imitators. He exhibited in France, Finland, and Japan. But he also experienced the downside of fame, along with financial and other difficulties. He fled Nigeria and traveled to the United States, where he settled for a while in Philadelphia. His work, again recognized and appreciated, is now found in museums around the world.

“I was doing it because that was what came from my mind,” the artist said referring to his subject matter, the unseen. His approach was syndetic, circuitous, and surprising. He started working on one end of the canvas and continued until every bit of the surface was covered, leaving the impression that more could always be added. He reveled in complexity and used it to engage the viewer. “I want to make a picture where the more you look, the more you see.” Still and always a musician, he found a rhythm and danced to it, creating dynamic and repetitive patterns in a seemingly endless continuous design. “It is helpful to have something else to do after you’ve made a mistake,” he advised. “You come back, and the mistake is fixed. Your subconscious will solve it.” Continuous engagement opens up the world.

The title *The Lazy Hunters, and the Poisonous Wrestlers, Lizard Ghost and the Cobra* of the painting on this month’s cover suggests a scene from some Yoruba tale but offers no further explanation. Like the plot in a folk opera with spoken parts along with music and dance, such as some that the artist himself may have participated in, it involves the common people, hunters, bumping into the extrahuman in the “bush of ghosts.” They are clearly at a disadvantage as they huddle in the corner under the serpent in the ferocious lizard claws of his imagination, affirming that African myths with animals are often about the strengths or foibles of people.

The farming town of Oshogbo was likely founded in the early 18th century by hunters from a nearby village languished by famine. Details are scant and mixed with legendary deals with local deities to secure water and leadership rights. In a culture that rewarded self-reliance and judged its members by their achievements, hunters had to survive in the forest, fully expecting to take on the role of warrior or scout in time of conflict. Yet in Twins’ version the hunters are branded lazy and appear powerless, their implements strewn in the foreground. Tiny against the animals, they seem frozen in fright, wild-eyed, and panic stricken.

Stacked on the left are dwellings, framing a community in the background. Nature is present in elusive forms since everything is woven with the same thread. Lizard fins double as mountains, flowers as oracles, beast attire as staring eyelets. The inflated lizard and serpent, an extraordinary spectacle, are inhabited by ancestral or other spirits. The scene has the static glow of a dream, but one thing is certain. Humans here are not the dominant species. They have lost control of the village to the lizard and cobra now locked mouth-to-mouth in a poisonous embrace.

Twins’ vision cuts through daily realities to expose a most inclusive web of interconnectedness. And through myth he gently navigates the web, guiding the viewer. He unveils mysterious forces unleashed in the forest. And while the beasts wrestle, he delivers more about this struggle than meets the eye.

Magic and medicine share the same name in Yoruba language and both rely on natural objects with supernatural powers to prevent or cure disease, which occurs when a person’s relationship with nature is disrupted. A balanced relationship is critical to life and health. Whether linked to twinning and infant deaths or to novel viruses and other causes of infection, the danger can become inflated out of proportion or curbed with public health measures. Having been tossed back and forth so many times, Twins rightly views himself as one charged with making images here from the other world and calling attention to lapses in vigilance.
